# Pembrolizumab-Related Sclerosing Cholangitis in a Patient With High Microsatellite Instability Gastric Cancer: A Case Report

**DOI:** 10.7759/cureus.66425

**Published:** 2024-08-08

**Authors:** Shusaku Honma, Satoshi Watanabe, Sanae Nakajima

**Affiliations:** 1 Surgery, Kobe City Medical Center West Hospital, Kobe, JPN; 2 Gastroenterology, Kobe City Medical Center West Hospital, Kobe, JPN

**Keywords:** immune-related liver injury, pembrolizumab, immune-related sclerosing cholangitis, immune-related adverse event (irae), advanced gastric cancer

## Abstract

Pembrolizumab is widely used to treat various malignant tumors, including gastric cancer; however, it is associated with immune-related adverse events. Among these adverse events, immune-related sclerosing cholangitis (irSC) is a rare type induced by pembrolizumab, and much remains unknown regarding its clinicopathological features and ideal management. Herein, we report the case of a 67-year-old man who received pembrolizumab for postoperative lymph node recurrence of gastric cancer. He developed irSC after the 15th course of pembrolizumab monotherapy, which was diagnosed using radiological imaging and liver biopsy. The patient was successfully treated with prednisolone. Eight months after the onset of irSC, the dose of prednisolone was tapered, and computed tomography revealed that the treatment response to pembrolizumab was maintained with progression-free lymph node metastasis despite not receiving any anticancer treatment. Understanding the characteristic imaging findings and clinicopathological features of irSC is crucial. Further accumulation of cases is necessary to establish the optimal management of irSC and to identify biomarkers that predict its risk.

## Introduction

Immune checkpoint inhibitors (ICIs) have recently been widely used to treat various malignant tumors. In the randomized phase III CheckMate649 and ATTRACTION-4 trials for advanced gastric cancer, nivolumab plus chemotherapy improved progression-free survival (PFS) and overall survival rates compared with chemotherapy alone [1,2]. In the nonrandomized phase II KEYNOTE-158 trial for advanced non-colorectal cancer with a deficiency in DNA mismatch repair or high microsatellite instability (MSI-high), pembrolizumab monotherapy showed an overall response rate of 45.8% and a median PFS of 11.0 months in 24 patients with gastric cancer [3]. Based on these results, the Japanese Gastric Cancer Treatment Guidelines 2021 recommend nivolumab plus chemotherapy as the first-line treatment for advanced gastric cancer and pembrolizumab monotherapy as the second-line treatment for patients with MSI-high.

Pembrolizumab, a humanized immunoglobulin G4 monoclonal antibody against human programmed cell death-1 (PD-1), activates tumor-specific cytotoxic T cells by inhibiting the binding of PD-1 to its ligands [4]. Among patients with gastric cancer, 5-22% have been reported to have MSI-high [5-7]. There have been reports that pembrolizumab monotherapy has a significant effect on advanced gastric cancer [8,9]. Although pembrolizumab monotherapy is a promising treatment for MSI-high gastric cancer, its use is associated with immune-related adverse events (irAEs) that may cause life-threatening side effects. irAEs occur in a variety of organs and can cause interstitial pneumonia, enterocolitis, hepatitis, skin rash, hypothyroidism, type 1 diabetes, or myocarditis; however, immune-related sclerosing cholangitis (irSC) is a rare type of irAE, and its clinicopathological features and ideal management remain to be established [10,11]. Herein, we report a case of irSC in a patient who was treated with pembrolizumab for advanced gastric cancer.

## Case presentation

**Figure 1:**
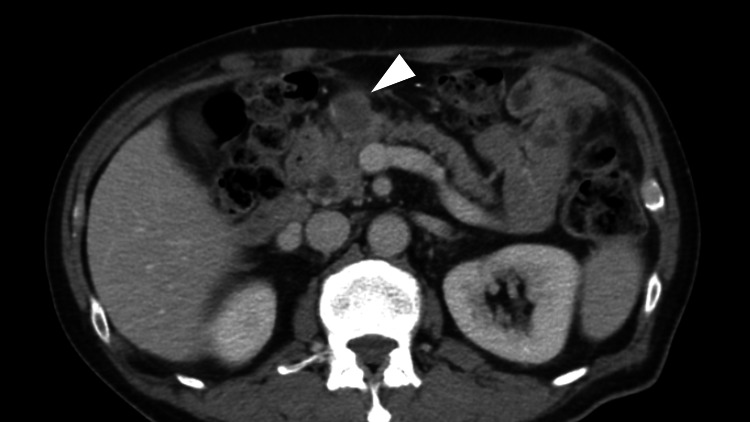
Axial abdominal contrast-enhanced computed tomography six months after laparoscopic distal gastrectomy An enlarged lymph node (arrowhead) is observed in front of the pancreas.

**Figure 2:**
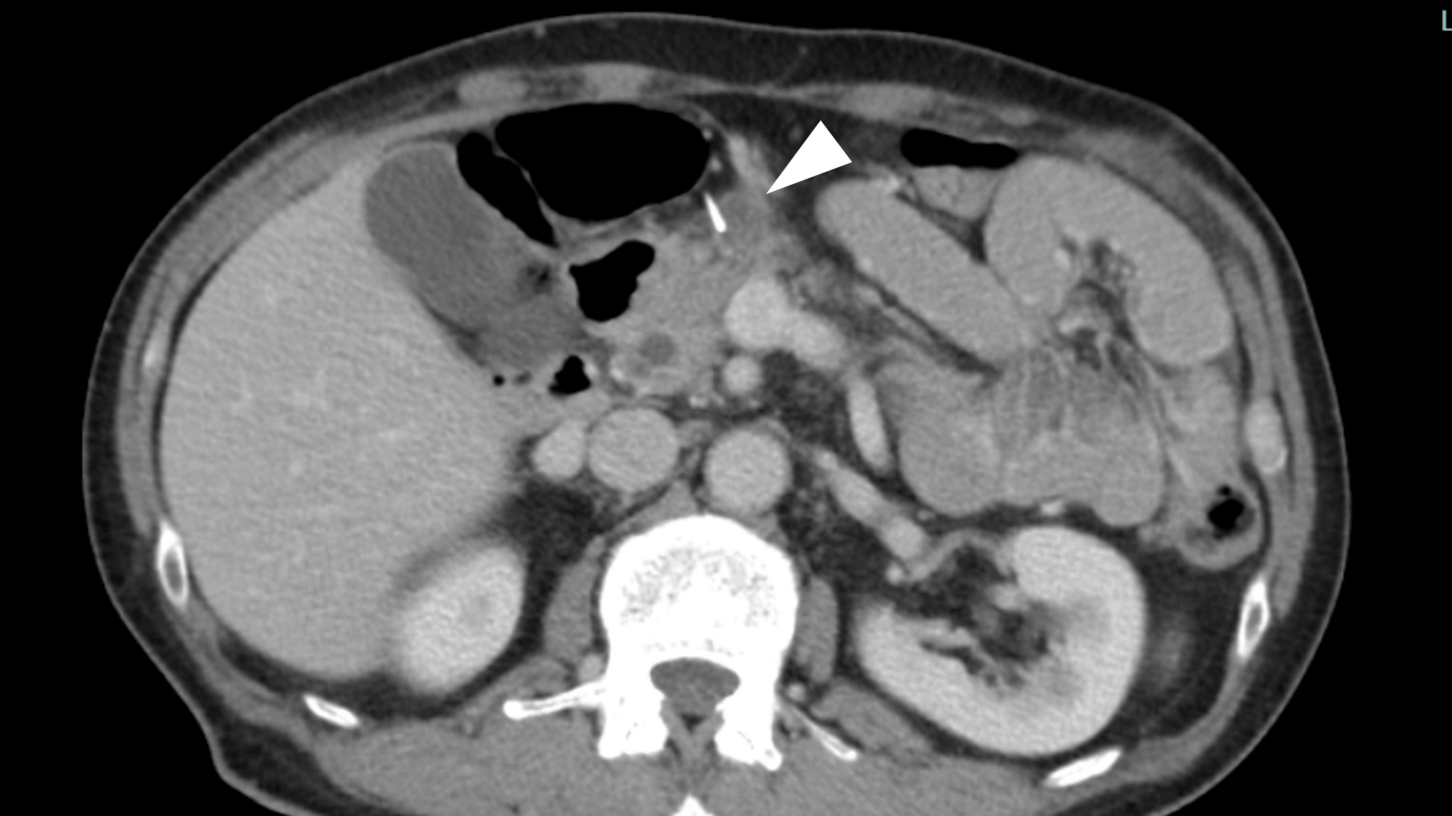
Axial abdominal contrast-enhanced computed tomography after four courses of pembrolizumab monotherapy Lymph nodes (arrowhead) shrank after four courses of pembrolizumab monotherapy.

**Figure 3:**
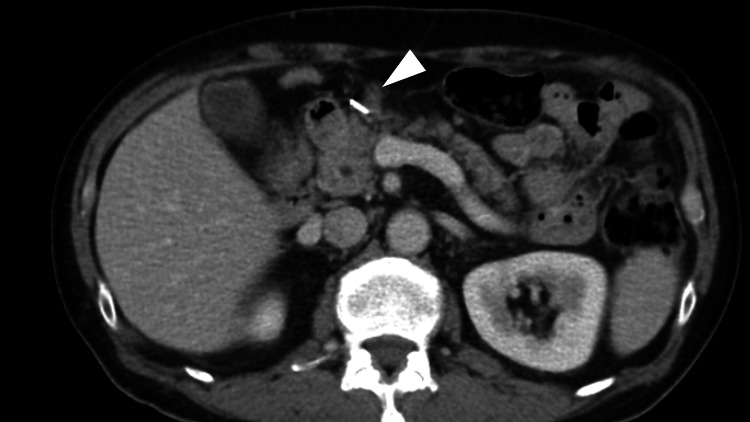
Axial abdominal contrast-enhanced computed tomography after 13 courses of pembrolizumab monotherapy The enlarged lymph nodes (arrowhead) almost disappeared after 13 courses of pembrolizumab monotherapy.

A 67-year-old man, who had undergone laparoscopic distal gastrectomy for pathological T3N3aM0 gastric adenocarcinoma according to the eighth edition of the UICC-TNM classification, followed by adjuvant chemotherapy with docetaxel and S-1 for five months, developed lymph node metastasis six months after surgery. Tumor marker levels were normal for carcinoembryonic antigen (2.7 ng/mL) and carbohydrate antigen 19-9 (9.5 ng/mL). Chemotherapy was considered the second-line treatment for recurrence during adjuvant chemotherapy. Histopathological examination of the resected specimen indicated negative human epidermal growth factor receptor-2 (HER-2) and MSI-high statuses; therefore, the patient was administered pembrolizumab monotherapy (200 mg once every three weeks). After four courses, contrast-enhanced computed tomography (CECT) revealed that the lymph nodes had shrunk. A partial response was diagnosed based on the Response Evaluation Criteria in Solid Tumors guidelines, and pembrolizumab monotherapy was continued. After six courses, he developed a grade 2 rash that required topical steroids for two months. We diagnosed the rash caused by pembrolizumab as an irAE. After 13 courses, CECT revealed that the enlarged lymph nodes had almost completely disappeared (Figures 1-3).

**Figure 4:**
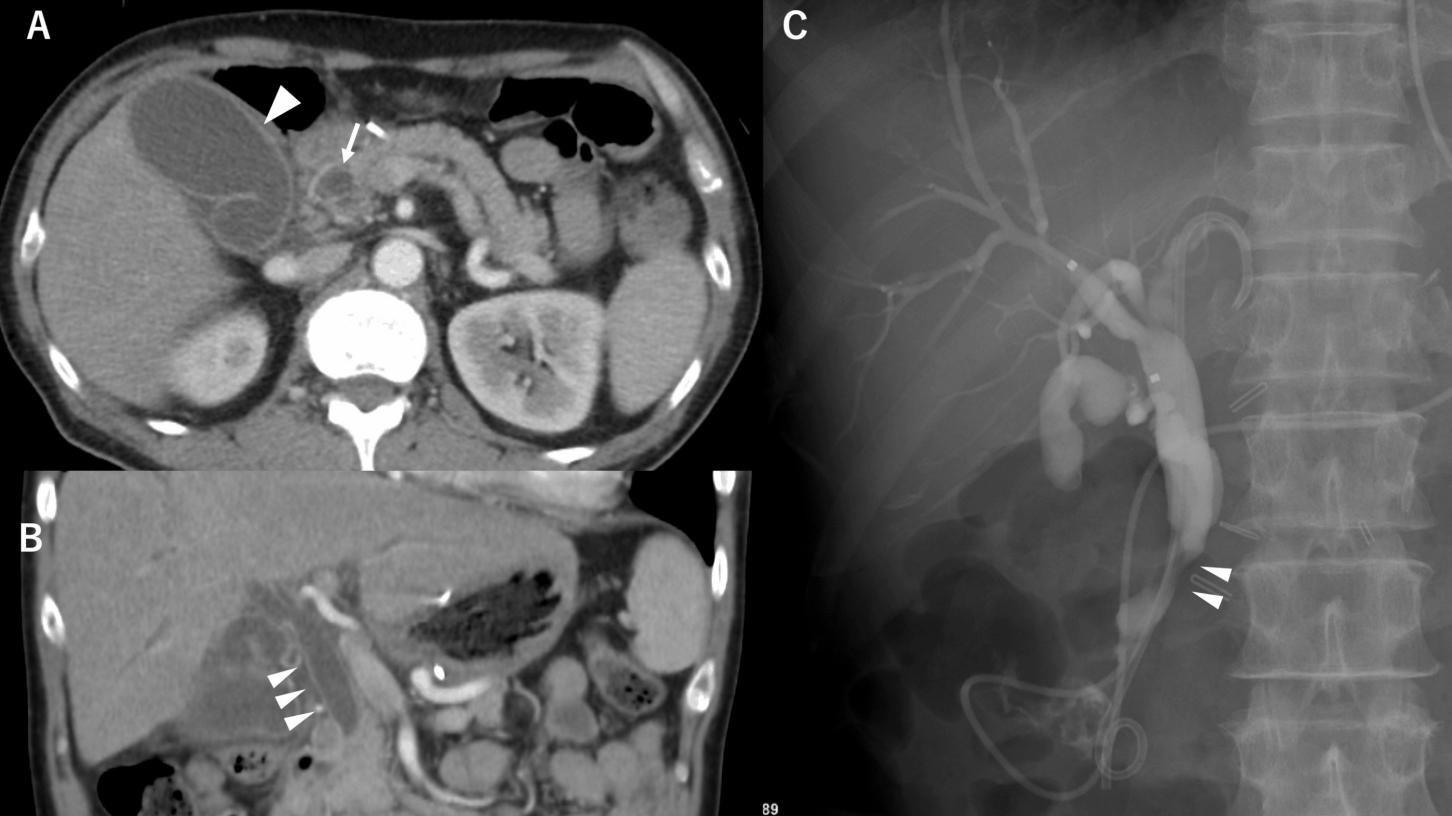
Clinical imaging findings at the time of immune-related adverse event cholangitis (A) Axial contrast-enhanced computed tomography (CECT) reveals the common bile duct (CBD) dilation (arrow) and diffuse gallbladder wall thickening (arrowhead). (B) Coronal CECT reveals the CBD dilation of up to 11 mm and diffuse CBD wall thickening (arrowheads). (C) Endoscopic retrograde cholangiopancreatography shows biliary tract dilation localized to the extrahepatic lesion with distal narrowing (arrowheads) and no obstruction.

Nine days after the 15th treatment course, the patient was hospitalized for general fatigue and fever. Physical examination revealed jaundice of the skin and conjunctiva. No abdominal pain was reported. Laboratory data revealed a marked increase in biliary enzymes: total bilirubin, 8.1 mg/dL (reference value, 0.1-1.0 mg/dL); direct bilirubin, 5.9 mg/dL (reference value, <0.2 mg/dL); gamma-glutamyl transferase, 336 IU/L (reference value, 12-70 IU/L); and alkaline phosphatase-International Federation of Clinical Chemistry, 455 IU/L (reference value, 38-113 IU/L), and a slight increase in hepatic enzymes: alanine aminotransferase, 111 IU/L (reference value, 5-36 IU/L); and aspartate aminotransferase, 79 IU/L (reference value, 9-35 IU/L). The white blood cell count was normal (3980 cells/μL (reference value, 3900-9800 cells/μL)), but C-reactive protein was increased to 5.34 mg/dL (reference value, <0.5 mg/dL). CECT revealed common bile duct (CBD) dilation and diffuse wall thickening of the CBD and gallbladder. There was no evidence of a tumor obstructing the CBD or metastatic lymph node progression. Endoscopic retrograde cholangiopancreatography (ERCP) revealed CBD dilation (Figures 4A-4C).

**Figure 5:**
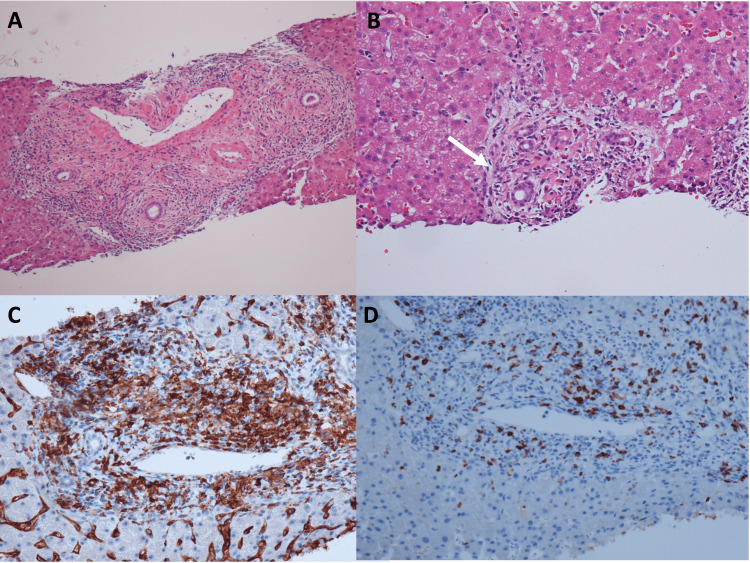
Pathological findings of liver biopsy (A) Hematoxylin and eosin staining: The portal tract is enlarged with inflammatory cell infiltration of lymphocytes, eosinophils, and plasmacytes (×100 magnification). (B) Hematoxylin and eosin staining: Disordered arrangement of the bile duct epithelium and hyperplasia of the bile canaliculi are observed (arrow). No bile embolism or granulomatous changes are observed (×200 magnification). (C) and (D) CD4 and CD8 staining, respectively: Numerous CD4 and CD8 positive cells are found in both the parenchyma and portal tract (×200 magnification).

Although retrograde biliary drainage tubes and nasobiliary drainage tubes were placed, bilirubin levels did not decrease. The serological test results for viral hepatitis and antibodies associated with autoimmune hepatitis, including antinuclear and antimitochondrial antibodies, were negative. The serum IgG level was normal (1096 mg/dL (reference value, 870-1700 mg/dL)), but the serum IgE level was markedly elevated (2000 mg/dL (reference value, <170 mg/dL)). We suspected pembrolizumab-related cholangitis as an irAE; therefore, we performed a percutaneous liver biopsy and administered hydrocortisone to the patient. The pathological findings of the liver biopsy were as follows: the portal tract was enlarged, with inflammatory cell infiltration by lymphocytes, eosinophils, and plasmacytes. A decrease in the bile ducts, disordered arrangement of the bile duct epithelium, and hyperplasia of the bile canaliculi were observed. Fibrosis and focal necrosis were mild, and no bile embolism or granulomatous changes were noted. Immunohistochemical staining for CD4 and CD8 showed that, although CD4+ cells were dominant, numerous CD8+ cells were found in both the parenchyma and portal tract (Figures 5A-5D).

**Figure 6:**
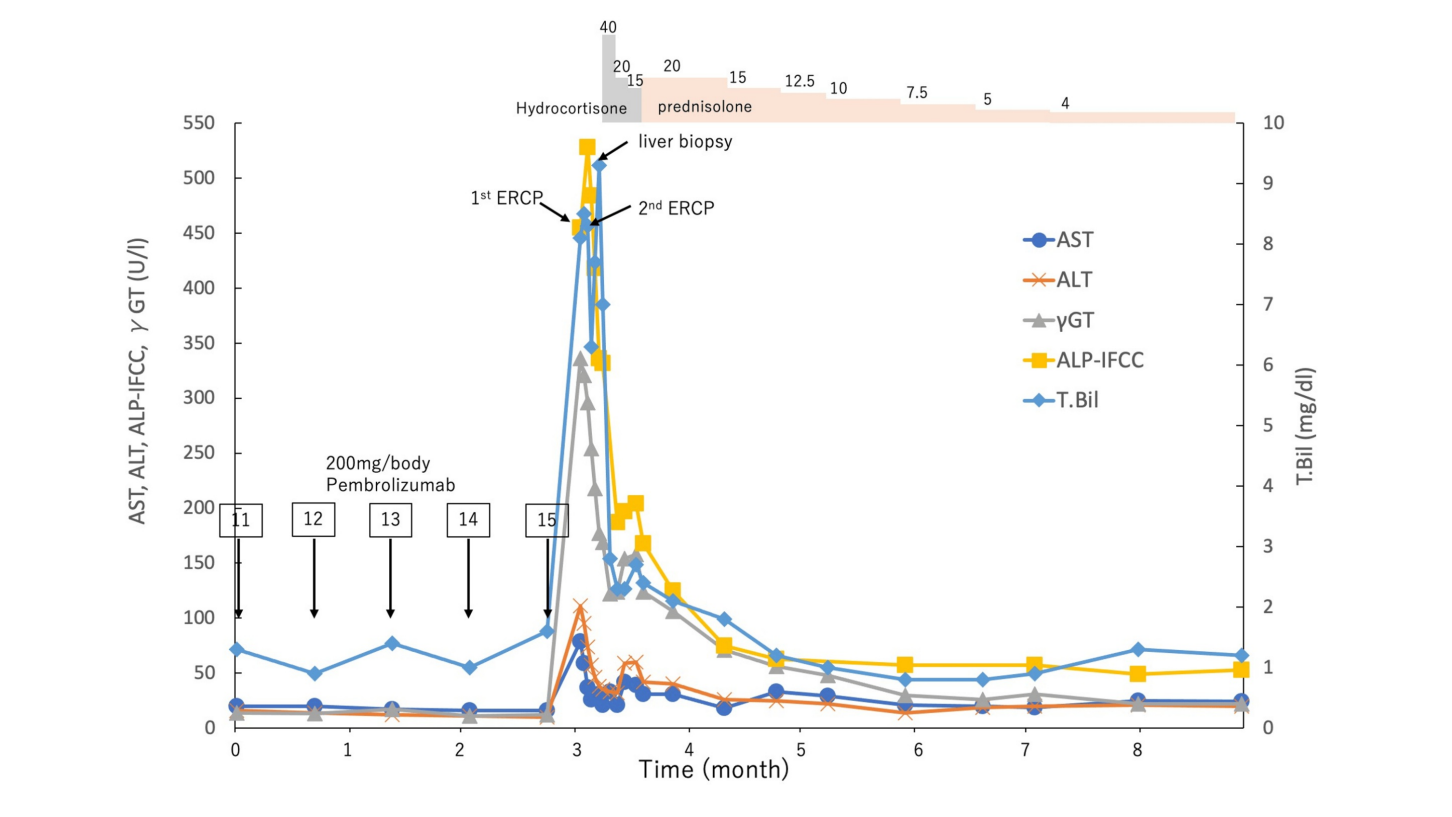
Summary of the clinical course and biochemical examinations ERCP, endoscopic retrograde cholangiopancreatography; T-Bil, total bilirubin; γ-GT, gamma-glutamyl transferase; AST, aspartate aminotransferase; ALT, alanine aminotransferase; ALP-IFCC, alkaline phosphatase-International Federation of Clinical Chemistry

These findings were consistent with the diagnosis of irSC. When the hydrocortisone dose was reduced to 15 mg/day, laboratory data worsened; therefore, prednisolone was administered as an alternative to hydrocortisone. Prednisolone was effective in immediately improving hepatobiliary enzymes (Figure 6).

**Figure 7:**
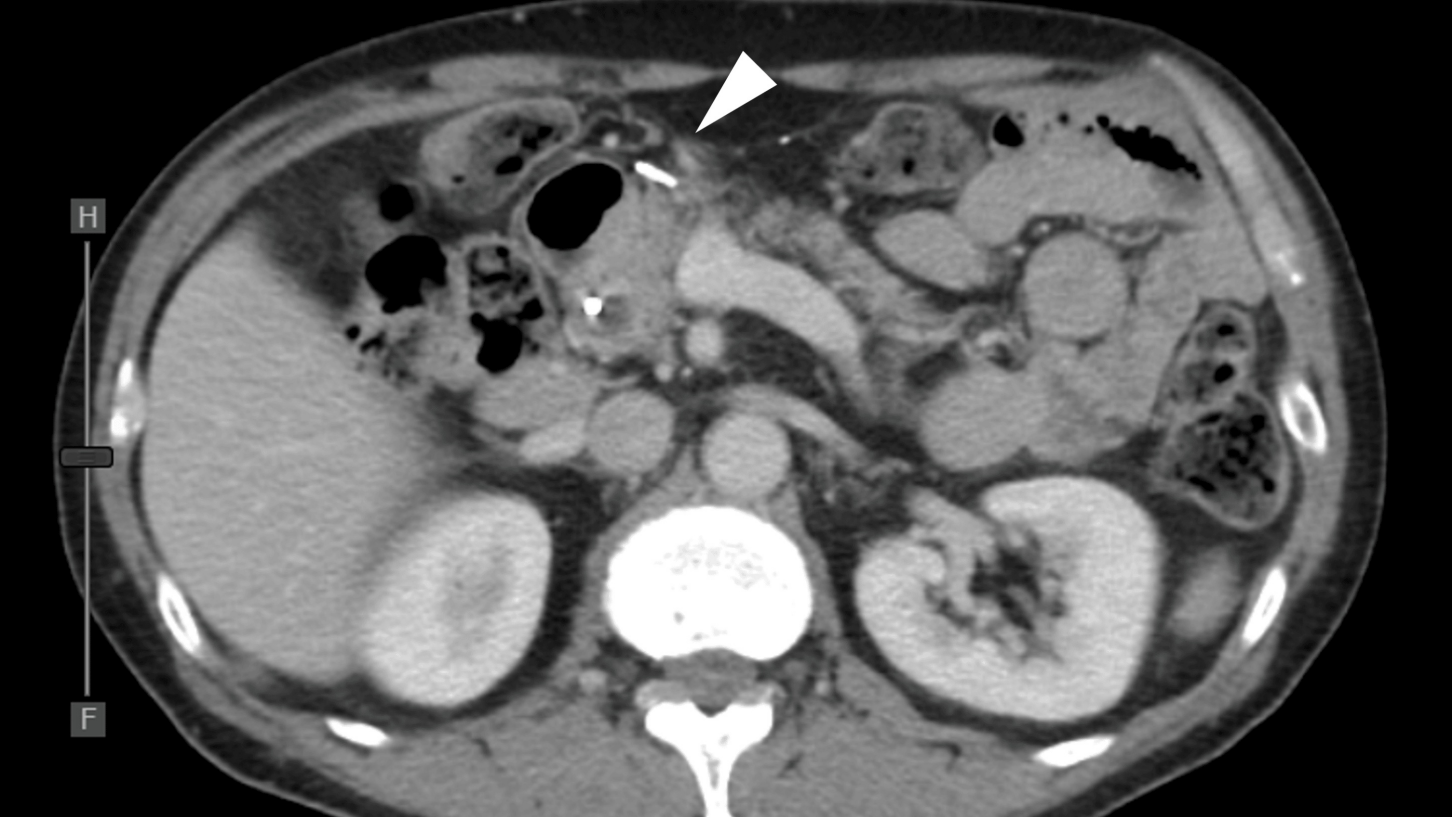
Axial imaging of abdominal contrast-enhanced computed tomography eight months after immune-related sclerosing cholangitis No re-enlargement of the lymph nodes is observed (arrowhead).

**Figure 8:**
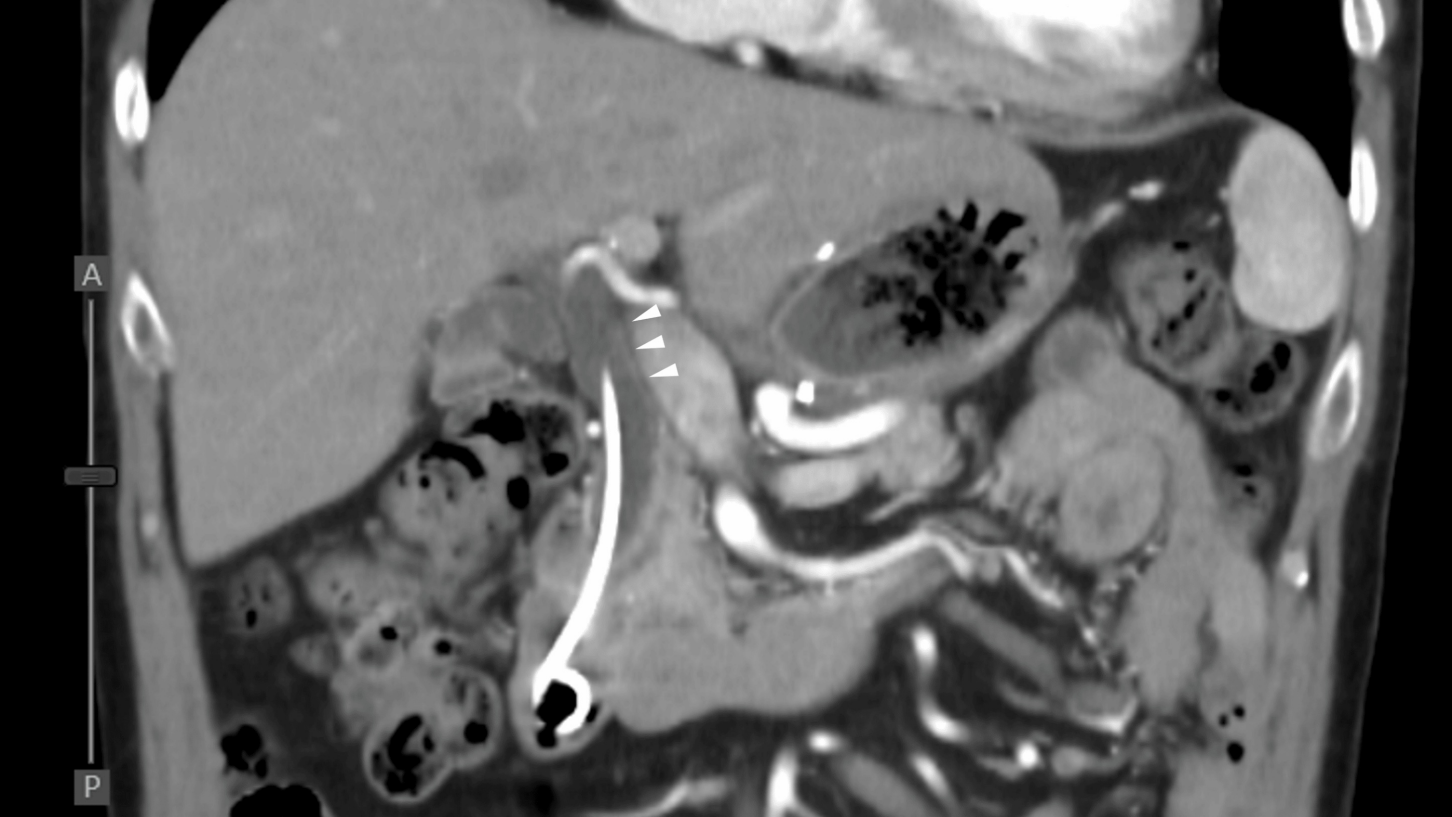
Coronal imaging of abdominal contrast-enhanced computed tomography eight months after immune-related sclerosing cholangitis Coronal imaging shows that although the common bile duct (CBD) dilation remained, diffuse CBD wall thickening improved (arrowheads).

Eight months after the onset of cholangitis, the patient was an outpatient with a good general health status. The prednisolone dose was successfully tapered down to 4 mg/day, and CT revealed that the treatment response was maintained with progression-free lymph node metastasis, despite not receiving any anticancer treatment (Figures 7-8). 

## Discussion

irSCs were first reported as adverse liver injuries caused by nivolumab [12]. Kawakami et al. also reported three cases of nivolumab-related cholangitis in patients with non-small cell lung cancer [13]. In that report, nivolumab-related cholangitis was characterized by: (I) localized extrahepatic bile duct dilation without obstruction; (II) diffuse hypertrophy of the extrahepatic bile duct wall; (III) a dominant increase in the biliary tract enzymes alkaline phosphatase and gamma-glutamyl transferase relative to the hepatic enzymes aspartate aminotransferase and alanine aminotransferase; (IV) normal or reduced levels of serum immunological markers, such as antinuclear antibody, antimitochondrial antibody, smooth muscle antibody, and immunoglobulin G4; (V) the pathological finding of biliary tract CD8+ T-cell infiltration on liver biopsy; and (VI) a moderate-to-poor response to steroid therapy. However, due to the small sample size of the report, PD-1 inhibitor-related sclerosing cholangitis remains unclear. In a systematic review of PD-1 inhibitor-related sclerosing cholangitis cases, Onoyama et al. evaluated the clinical and pathological features of 31 cases, including three cases of gastric cancer and 10 patients treated with pembrolizumab [14]. They found that the median number of cycles until the onset of PD-1 inhibitor-related sclerosing cholangitis was 5.5 (range, 1-27). They also reported biliary dilation without obstruction, diffuse hypertrophy of the extrahepatic biliary tract, and multiple strictures of the intrahepatic biliary tract in 76.9%, 90.5%, and 30.4% of patients, respectively [14]. They demonstrated that PD-1 inhibitor-related sclerosing cholangitis can be divided into three groups: (I) extrahepatic type, diffuse extrahepatic biliary hypertrophy without biliary stenosis; (II) intrahepatic type, multiple stenoses, especially in the intrahepatic bile duct, without extrahepatic biliary hypertrophy; and (III) diffuse type, diffuse biliary tract hypertrophy with multiple stenoses of the intrahepatic and extrahepatic bile ducts. Our case was similar to the extrahepatic type. The clinical significance of this classification is uncertain; however, it is expected to be defined with the accumulation of cases in the future.

With the approval of additional ICIs for several malignant tumors, it is important to predict irAEs to improve patient survival and quality of life [15]. A high neutrophil-to-lymphocyte ratio and an increase in C-reactive protein and serum interleukin-6 levels are considered predictors of irAEs; however, no single blood-based biomarker has been identified thus far [16]. In their investigation of 29 cases of liver injury, including four irSCs induced by ICIs in Japan, Mizuno et al. reported that ipilimumab use and fever within 24 hours of the initial ICI administration were risk factors for immune-related liver injury [11]. However, the management of irSCs remains unclear. Discontinuation of ICI is the first step in treating irSCs, similar to the treatment of all severe irAEs. In contrast to immune-related hepatitis, irSCs are typically steroid-resistant. The aforementioned systematic review demonstrated that only three of 26 patients showed improvement to normal levels of liver and biliary enzymes with steroid therapy [14]. They suggested that other medications, such as ursodeoxycholic acid, azathioprine, bezafibrate, and tacrolimus, are effective alternatives to steroid therapy in improving irSCs [14]. 

In our case, we decided to perform a follow-up without chemotherapy based on the patient’s request after overcoming the acute stage of irSCs. Some reports have shown that the occurrence of irAEs is associated with a better clinical outcome of ICIs in patients with malignant tumors, including advanced gastric cancer [17,18]. Our patient showed a relatively long PFS despite not receiving any anticancer treatment. Although permanent discontinuation of ICIs is generally recommended for grade 4 toxicities [19], a cohort study reported a 28.8% recurrence rate of the same irAEs associated with the discontinuation of ICI therapy after a rechallenge with the same ICI [20]. They concluded that resuming ICI therapy could be considered for select patients, with careful monitoring and the use of standard treatment algorithms to identify and treat toxic effects.

## Conclusions

Given the increasing use of pembrolizumab and other ICIs for treating various malignant diseases, understanding the characteristic imaging findings and clinicopathological features of irSCs is crucial. Further studies are needed to establish the optimal management of irSCs and to identify biomarkers that have the potential to accurately predict the risk of irAE development in patients undergoing ICI treatment.
